# Enhanced cardiovascular disease prediction through self-improved Aquila optimized feature selection in quantum neural network & LSTM model

**DOI:** 10.3389/fmed.2024.1414637

**Published:** 2024-06-20

**Authors:** Aman Darolia, Rajender Singh Chhillar, Musaed Alhussein, Surjeet Dalal, Khursheed Aurangzeb, Umesh Kumar Lilhore

**Affiliations:** ^1^Department of Computer Science and Applications, M.D. University, Rohtak, Haryana, India; ^2^Department of Computer Engineering, College of Computer and Information Sciences, King Saud University, Riyadh, Saudi Arabia; ^3^Department of Computer Science and Engineering, Amity University, Gurgaon, Haryana, India; ^4^Department of Computer Science and Engineering, Galgotias University, Greater Noida, Uttar Pradesh, India

**Keywords:** quantum neural network, cardiovascular disease prediction, feature extraction, self-improved Aquila optimization, machine learning

## Abstract

**Introduction:**

Cardiovascular disease (CVD) stands as a pervasive catalyst for illness and mortality on a global scale, underscoring the imperative for sophisticated prediction methodologies within the ambit of healthcare data analysis. The vast volume of medical data available necessitates effective data mining techniques to extract valuable insights for decision-making and prediction. While machine learning algorithms are commonly employed for CVD diagnosis and prediction, the high dimensionality of datasets poses a performance challenge.

**Methods:**

This research paper presents a novel hybrid model for predicting CVD, focusing on an optimal feature set. The proposed model encompasses four main stages namely: preprocessing, feature extraction, feature selection (FS), and classification. Initially, data preprocessing eliminates missing and duplicate values. Subsequently, feature extraction is performed to address dimensionality issues, utilizing measures such as central tendency, qualitative variation, degree of dispersion, and symmetrical uncertainty. FS is optimized using the self-improved Aquila optimization approach. Finally, a hybridized model combining long short-term memory and a quantum neural network is trained using the selected features. An algorithm is devised to optimize the LSTM model’s weights. Performance evaluation of the proposed approach is conducted against existing models using specific performance measures.

**Results:**

Far dataset-1, accuracy-96.69%, sensitivity-96.62%, specifity-96.77%, precision-96.03%, recall-97.86%, F1-score-96.84%, MCC-96.37%, NPV-96.25%, FPR-3.2%, FNR-3.37% and for dataset-2, accuracy-95.54%, sensitivity-95.86%, specifity-94.51%, precision-96.03%, F1-score-96.94%, MCC-93.03%, NPV-94.66%, FPR-5.4%, FNR-4.1%. The findings of this study contribute to improved CVD prediction by utilizing an efficient hybrid model with an optimized feature set.

**Discussion:**

We have proven that our method accurately predicts cardiovascular disease (CVD) with unmatched precision by conducting extensive experiments and validating our methodology on a large dataset of patient demographics and clinical factors. QNN and LSTM frameworks with Aquila feature tuning increase forecast accuracy and reveal cardiovascular risk-related physiological pathways. Our research shows how advanced computational tools may alter sickness prediction and management, contributing to the emerging field of machine learning in healthcare. Our research used a revolutionary methodology and produced significant advances in cardiovascular disease prediction.

## 1 Introduction

CVD is a global health issue that kills many people. The WHO estimates a 37% mortality rate, affecting 17.9 million people (1). CVD deaths are mostly caused by stroke and heart disease. These frightening findings highlight the need to understand the complex causes of CVD. The complex nature of CVD, which is linked to risk factors like high blood pressure, insulin levels, smoking, and sedentary lifestyles, highlights the need for comprehensive prevention, early detection, and management strategies (2). Understanding these risk variables is essential for establishing targeted therapies and reducing the global effect of cardiovascular health issues as researchers study CVD (3). Studies show that up to 90% of CVD cases are avoidable, but early detection, treatment, and recovery are crucial. Early CVD detection is essential for timely interventions. However, CVD prediction is too sophisticated for the brain. Time dependency, erroneous results, and knowledge upgradation due to vast CVD datasets complicate identification (4). These datasets typically have irrelevant and redundant features that hamper classification. Noise from unwanted features affects system performance. Addressing this, our research focuses on FS to eliminate unwanted features before applying classification approaches. This process enhances model simplification, reduces the risk of overfitting, and improves computational efficiency (5).

Traditional diagnosis heavily relies on clinical signs and symptoms, making disease analysis challenging. Predicting CVD is particularly complex due to multiple contributing factors, leading to inconsistent outcomes and assumptions. In the medical domain, data mining (DM) methods, especially ML techniques (6), are employed to analyze diseases like cancer, stroke, diabetes (7), and CVD. This research specifically utilizes advanced DM approaches for studying CVD. Also, some more accurate DM approaches are being used to study heart disease. Researchers have applied various DM systems such as support vector machines (SVM), decision trees (DT), and artificial neural networks (ANN) to identify CVD (8). With all of the above methods, patient records are continuously categorized and predicted. It continuously checks the patient’s movements and informs the patient and doctor of the risk of illness if there is a change. With the help of techniques like ML, doctors can easily detect CVD in the early stage itself. Amongst the traditional invasive-based method, angiography is represented as the well-known heart problem diagnosis method but, it has some limits. Conversely, a method such as intelligent learning-based computational approaches, non-invasive-based techniques is considered more effective for predicting CVD. Cardiovascular disease (CVD), one of the leading causes of death worldwide, causes much morbidity and death. Early detection and prediction are essential to prevent CVD and reduce its impact on individuals and healthcare systems. Medical advances in machine learning and predictive analytics have created promising new opportunities for early cardiovascular disease risk factor diagnosis (9).

Predicting cardiovascular disease is crucial due to its incidence and damage. High-risk patients can be identified, advised on lifestyle changes, and prevented from developing cardiovascular disease (CVD). Genetic and risk factor-based predictive diagnostics provide individualized healthcare and tailored medicines. Traditional risk assessment and advanced machine learning algorithms predict cardiovascular disease. Traditional risk assessments like the Framingham Risk Score and Reynolds Risk Score use demographic, clinical, and biochemical data to estimate CVD risk across time. These techniques have directed primary preventive initiatives by identifying high-risk populations. Machine learning algorithms’ ability to search massive data sets for detailed patterns has propelled their rise in cardiovascular disease prediction. More accurate and powerful predictive models have been constructed combining electronic health records, imaging data, genetic information, and lifestyle factors using supervised learning approaches such logistic regression, support vector machines, random forests, and neural networks. Before predictive analytics can fully forecast cardiovascular illness, many challenges must be overcome. Multiple data sources, such as genetic data, wearable sensor data, and socioeconomic characteristics, make cohesive prediction models difficult. Integrating all these data types while maintaining privacy, interoperability, and quality is still difficult. When clinical decision-making is crucial, machine learning model interpretability is a concern. Black-box algorithms can produce accurate predictions, but healthcare practitioners are wary of them since they do not expose their inner workings. Because cardiovascular disease risk changes, models must be developed and validated for varied populations and healthcare systems (10).

Future multidisciplinary teams of medics, data scientists, and AI professionals will improve cardiovascular disease prediction. Integrating data from microbiomics, proteomics, metabolomics, and genomes may lead to new cardiovascular risk biomarkers and better risk prediction models. Wearables, smartphone health apps, and remote monitoring systems enable real-time risk assessment and personalized treatments based on lifestyle and physiological parameters. Here, a Hybrid Intelligent Model with an Optimal Feature Set is introduced for the prediction of CVD. The main contributions are summarized below:

1. The proposed research addresses the issue of dimensionality reduction by implementing FS techniques to reduce the number of features.
2. To introduce the SIAO method for optimal FS, overcoming challenges in extensive CVD datasets.
3. Proposing a hybrid model that combines LSTM and QNN to enhance the prediction performance of CVD.

The subsequent sections follow a structured framework: Section 2 reviews conventional CVD prediction models. In Section 3, the proposed model architecture is presented, and discussions on feature extraction, central tendency, dispersion, qualitative variation, and symmetrical uncertainty are provided. Section 4 introduces SIAO for optimal FS. The hybrid LSTM-QNN classification method is covered in Section 5. Experimental results and discussions are presented in Section 6. Section 7 contains the conclusion, summarizing contributions, and suggesting future research.

## 2 Literature review

This section critically analyses CVD prediction approaches, highlighting significant research and their contributions to the discipline. Using an Improved Quantum CNN (IQCNN) for accuracy, Pitchal et al. (11) developed an automated model for heart disease prediction that includes preprocessing, feature extraction, and prediction. This technique, which surpassed Bi-LSTM and CNN with 0.91 accuracy, shows promise for using IoT technologies for health diagnosis. Innovative computer methods improve cardiac disease prediction in their work.

Li et al. (12) used a hybrid deep learning (DL) model to predict CVD. The hybrid model, which uses 7,291 patient data and two deep neural network (DNN) models and one RNN for training, outperformed standard methods in prediction accuracy. Secondary training with a kNN model improved predicted accuracy. Prediction accuracy of 82.8%, precision of 87.08%, recall of 88.57%, and F1-score of 87.82% in the test set outperform single-model ML predictions. The hybrid model reduced overfitting, improving CAD prediction and clinical diagnosis. Singh et al. (13) examined how IoMT devices transformed continuous CVD patient monitoring. Their study proposed an advanced DL framework for the IoMT ecosystem that could improve patient care by predicting CVD. They effectively extract spatial and sequential characteristics from diverse IoMT data sources, such as pulse oximeters and electrocardiograms, using their innovative hybrid CNN-RNN architecture. With the utilization of transfer learning (TL) and real-world data, the proposed model surpasses previous methods in terms of precision and resilience. Their research assists medical professionals in gaining insights into predictive factors, enhancing the model’s ability to be understood and its impact on therapy.

In their study, Oyewola et al. (14) utilized an ensemble optimization DL method to diagnose early CVD. They employed the Kaggle Cardiovascular Dataset for both training and testing purposes. The ensemble model achieves superior performance compared to neural network architectures, boasting an impressive accuracy rate of 98.45%. The research examined and provided a practical solution to streamline CVD diagnosis for doctors. It showcased the model’s impressive speed and precision in identifying patients and interpreting CVD test results, leading to advancements in healthcare practices. Incorporating wearable systems, exploring advanced ensemble techniques, and utilizing diverse data sources have been found to enhance predictive capabilities and improve model performance in real-world healthcare settings, according to recent research. In 2023, a team of researchers developed a cutting-edge model for assessing the risk of cardiovascular disease (CVD). They utilized advanced algorithms and optimization strategies to create the SOLSSA-CatBoost model, which shows great promise in this field. Their approach proved to be highly effective, surpassing the performance of multiple machine learning models and optimization techniques on Kaggle CVD datasets. They achieved impressive F1-scores of 90 and 81.51%. This work contributes to the field of predictive healthcare by offering a more precise tool for assessing the risk of cardiovascular disease. However, further research is required to evaluate its practicality and effectiveness in diverse populations.

In their study, Palanivel et al. (15) discussed the global health concern of cardiovascular disease (CVD) and emphasized the importance of early prediction. They presented a compelling approach that combines FS and an innovative Multi-Layer Perceptron (MLP) for Enhanced Brownian Motion based on Dragonfly Algorithm (MLP-EBMDA) classification using DM methods. This contribution encompasses an optimized unsupervised feature selection technique, a distinctive classification model with an accuracy of 94.28%, and a methodical approach to predicting early cardiovascular disease. The methodology is meticulously organized and precise, but it requires validation and real-world implementation.

In their study, Yewale et al. (16) devised a comprehensive framework for predicting cardiovascular disease. They made a deliberate choice to exclude FS and instead focused on data balance and outlier identification. Their work involved utilizing the Cleveland dataset to investigate various performance factors and achieve an impressive accuracy rate of 98.73% and sensitivity rate of 98%, surpassing previous research findings. The methodology demonstrates an impressive level of precision, with a specificity of 100%, positive prediction value of 100%, and negative prediction value of 97%. It also implemented OD by using a separate forest for a thorough analysis. Their work is notable for its meticulous evaluation metrics.

In their study, Behera et al. (17) devised a novel approach combining machine learning techniques to predict heart and liver diseases. They utilized a modified particle swarm optimization (PSO) algorithm in conjunction with support vector machines (SVM). The study focused on the rising occurrence of heart and liver disorders and the importance of promptly detecting them for better patient outcomes. By integrating SVM with modified PSO, the hybrid model achieved significant improvements in classification accuracy, error reduction, recall, and F1-score. The research’s empirical foundation is strengthened by the data from the UCI ML collection. In their study, Sudha and Kumar (18) proposed a hybrid CNN and LSTM network for predicting cardiovascular disease, aiming to tackle the pressing issue of timely and accurate detection on a global scale. Utilizing cutting-edge DL advancements, the suggested model seamlessly combined CNN and LSTM to surpass the accuracy limitations of traditional ML methods. The hybrid system achieved an accuracy of 89% on a CVD dataset following 10 k-fold cross-validation. The suggested analysis outperformed SVM, Naïve Bayes (NB), and DT models in terms of performance. Their approach stands out with its distinctive technique, impressive precision, and practicality as an alternative to ML models in predicting CVD.

Elavarasi et al. (19), provided a summary of the recent challenges in predicting cardiovascular disease (CVD), focusing on the issues faced by traditional systems and the complexity of deep learning (DL). They utilized the elephant search algorithm (ESA) to explore innovative interpretability solutions during their investigation. ESA is seamlessly integrated with SVM to enhance the accuracy of CVD prediction, even though it faces challenges when dealing with large datasets and computational complexity. They strive to enhance FS by enhancing the accuracy and interpretability of CVD dataset. Their research enhanced clinical decision support systems (DSSs), shedding light on the ongoing debate surrounding CVD prediction methodologies.

Table 1 summarizes standard CVD prediction models’ features and drawbacks. An Automated IQCNN Model improved heart disease prediction and IoT diagnostics (11), however dataset specificity and scalability were issues. Wei et al. (20)’s SOLSSA-CatBoost Model improved CVD risk assessment accuracy through algorithmic fusion, however real-world applicability was questioned. In Palanivel et al. (15), the MLP-EBMDA classification model showcased optimized unsupervised FS, a novel classification model with higher accuracy, and a systematic approach to early CVD prediction. Li et al. (12), proposed a hybrid DL model with features like the utilization of two DNN models and an RNN, achieving average accuracy and effectively addressing overfitting challenges. Singh et al. (13), introduced an IoMT-Enhanced DL framework, incorporating a hybrid architecture combining CNNs and RNNs, extracting spatial and sequential features from heterogeneous IoMT data sources, and emphasizing interpretability and impact on treatment processes. Oyewola et al. (14) proposed an ensemble optimization DL technique that stands out for outperforming various NN architectures with high accuracy and simplifying CVD diagnosis for medical professionals. Elavarasi et al. (19) presented an ESA-integrated SVM for CVD prediction, focusing on interpretability through FS and optimizing FS using ESA and SVM while addressing challenges associated with traditional systems. Yewale et al. (16), ensemble techniques with data balancing and OD achieved higher accuracy and sensitivity, demonstrating high specificity and positive prediction value, although a need for a diverse composition of metrics was identified. Behera et al. (17) proposed a hybrid ML algorithm incorporating PSO and SVM showcased the utilization of modified PSO-SVM, resulting in average classification accuracy and error reduction, with a call to investigate runtime complexity. Finally, Sudha and Kumar (18) proposed a hybrid CNN and LSTM CVD prediction approach with great accuracy proven by 10 k-fold cross-validation and recommended for real-world applications. These systematic reviews shed light on these models’ strengths and weaknesses, leading to CVD prediction methodology development between paragraphs belonging to the same section.

**Table 1:** Review of features and challenges of conventional models based on a prediction of CVD.

References	Deployed model	Features	Challenges
Pitchal et al. (11)	Automated IQCNN Model	Incorporates preprocessing, feature extraction, and prediction with IQCNN; Notable high accuracy level; Advances IoT use in health diagnostics	Reliance on specific datasets; Scalability in diverse healthcare settings
Li et al. (12)	Hybrid DL model	Utilizes two DNN models and an RNN; Achieved Average accuracy, precision, recall, and F1-score	Effectively addresses overfitting challenges
Singh et al. (13)	IoMT-Enhanced DL framework	Hybrid architecture combining CNNs and RNNs; Extracts spatial and sequential features from heterogeneous IoMT data sources; Incorporates TL and real-world data	Ensuring interpretability and impact on treatment processes
Oyewola et al. (14)	Ensemble Optimization DL technique	Outperforms various NN architectures with higher accuracy; Simplifies CVD diagnosis for medical professionals	Use of sophisticated ensemble techniques
Palanivel et al. (15)	MLP-EBMDA classification	Optimized unsupervised FS; Novel classification model with high accuracy; Systematic approach to early CVD prediction	Use more datasets to get accurate results.
Yewale et al. (16)	Ensemble techniques with data balancing and OD	Achieves High accuracy and sensitivity; Demonstrates High specificity and positive prediction value	Need to use a diverse composition of metrics.
Behera et al. (17)	Hybrid ML algorithm with PSO and SVM	Utilizes modified particle swarm optimization and SVM; Showcases Average classification accuracy and error reduction	Need to investigate the runtime complexity.
Sudha and Kumar (18)	Hybrid CNN and LSTM Network	Combines CNNs with LSTM networks for CVD prediction; Achieves High accuracy validated through k-fold cross-validation	Apply the hybrid approach to real-world applications
Wei et al. (20)	SOLSSA-CatBoost Model	Integrates improved SSA with CatBoost; Enhanced by salp swarm algorithm, OBL, and lateral mutation; Superior F1-scores	Real-world applicability and diverse population performance
Elavarasi et al. (19)	ESA-integrated SVM	Addresses challenges with traditional systems; Focuses on interpretability through FS; Optimizes FS using ESA and SVM	Handling large datasets and computational complexity

In essence, our proposed model, as outlined, integrates the strengths of DL, and bio-inspired algorithms techniques while systematically addressing the limitations identified in the existing approaches. The innovative features of our model, including optimized FS through SIAO and the hybridization of LSTM and QNN, contribute to its potential to provide enhanced accuracy, efficiency, and practical applicability in real-world CVD prediction scenarios.

## 3 Methodology

**Figure 1:**
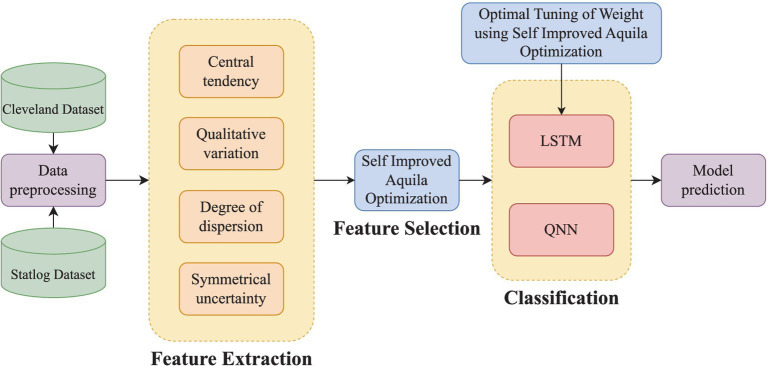
Proposed approach of CVD prediction.

The hybrid model averages LSTM and QNN classifier outputs to predict. The SIAO method optimizes LSTM weight adjustment, improving prediction model accuracy. Thus, CVD prediction works. As shown in Figure 1, CVD prediction involves four key steps: preprocessing, feature extraction, FS, and prediction.

- *Step 1: Preprocessing* – The initial stage removes duplicates and missing data to ensure data quality and dependability for analysis.
- *Step 2: Feature Extraction* – This phase involves detailed feature extraction. Central tendency, qualitative variation, dispersion, and symmetrical uncertainty are identified. These attributes help solve the dataset’s high dimensionality problem.
- *Step 3: Feature Selection* – The Symmetrical Uncertainty-based Iterative Algorithm Optimization (SIAO) technique is used to choose features optimally. This smart selection procedure improves model efficiency and accuracy by using only the most important features.
- *Step 4: CVD Prediction* – A hybrid model combining LSTM and QNN technology is trained using ideally selected features. This stage optimizes LSTM model weights using the SIAO algorithm. This optimization technique improves the model’s predictive power.

The proposed CVD prediction approach is shown in Figure 1.

### 3.1 Optimal selection of features via self-improved Aquila optimization

The extracted features pose challenges related to dimensionality reduction, prompting the utilization of an SIAO Algorithm for optimal FS in this research endeavor.

#### 3.1.1 SIAO algorithm

In 2021, Abualigah et al. (1) proposed an Aquila Optimization (AO), which is a modern swarm intelligence (SI) algorithm. Aguila consists of 4 types of hunting behaviors for specific sorts of prey. Aquila adeptly adapts hunting strategies for specific prey, utilizing its rapid velocity and robust talons; correspondingly, the AO Algorithm comprises four intricately designed stages as follows:

Expanded Exploration ( $X_{1}$ ): Excessive ascend with a vertical bend. Eq. 1 and Eq. 2 define the mathematical expression of expended exploration of AO, in which Aquila flies excessively over the floor and explores the quest area widely, and then a vertical dive can be taken as soon as the Aquila identifies the prey’s location.

(1) $$X_{1}\left(t+1\right)=X_{\mathrm{best}}\left(t\right).\left(1-\frac{1}{t}\right)+\left(X_{M\left(t\right)}-X_{\mathrm{best}}\left(t\right).\mathrm{rand}\right)$$

(2) $$X_{M}\left(t\right)=\frac{1}{N}\overset{N}{\underset{i=1}{\sum }}X_{i}\left(t\right)$$

Where better position attained was represented as $X_{\mathrm{best}}\left(t\right)$ and $X_{M\left(t\right)}$ represents the mean position of Aquila in the present iteration. t denoted as the current iteration and the T represents the maximum iteration. The size of the population is mentioned as N and a random number (between 0 and 1) is indicated as rand.

Narrowed Exploration ( $X_{2}$ ): Outline flight with the brief skim attack. Narrowed exploration is one of the frequently used hunting approaches for Aquila Employing brief gliding maneuvers for targeted prey attacks, the AO Algorithm elegantly combines sliding within the selected area and precise aerial navigation around the prey, with the refined exploration process succinctly defined by Eq. 3.

(3) $$X_{2}\left(t+1\right)=X_{\mathrm{best}}\left(t\right).LF\left(D\right)+X_{R\left(t\right)}+\left(y-x\right).\mathrm{rand}$$

Where Hawks’ random position is indicated as $X_{R\left(t\right)}$ , and the size of a dimension is denoted as D. Function of Levy flight $LF\left(D\right)$ , is expressed in below Eq. 4a and Eq. 4b.

(4a) $$LF\left(D\right)=s\times \frac{u\times \sigma }{{\left|v\right|}^{\frac{1}{K}}}$$

(4b) $$\sigma =\left[\frac{\Gamma \left(1+K\right)\times \sin\left(\frac{\pi K}{2}\right)}{\Gamma \left(\frac{1+K}{2}\right).K.2^{\left(\frac{K-1}{2}\right)}}\right]$$

Where Γ and K means stable values equivalent to 0.01 & 1.5; u and v denote random values between 0 & 1; y and x represent the spiral shape in the search. These values are mathematically calculated as follows (See Eq. 5):

x=r×sin∅

y=r×cos∅

$$r=r1+0.00565\times D_{1}$$

(5) $$\emptyset =-\omega \times D_{1}+\frac{3\times \pi }{2}$$

Where, the search cycle number is represented as r1 , which exists between the range of 1 and 20, the value of ω is equal to 0.005. Also, $D_{1}$ is mentioned as the integer values and D indicates the size of the dimensions.

Extended Exploitation ( $X_{3}$ ): Executing the minimal flight strategy with a calibrated descent attack, the Aquila adopts a nuanced approach. In this tactical maneuver, the prey’s location is approximately ascertained, prompting the Aquila to initiate a vertical assault. The AO algorithm strategically capitalizes on the identified region, meticulously navigating closer to the prey before launching the attack. This intricate behavior is mathematically articulated in Eq. 6.

(6) $$\begin{array}{l} X_{3}\left(t+1\right)=X_{\mathrm{best}}\left(t\right)-X_{M}\left(t\right).\alpha -r\\ \mathrm{and}\left(\left(UB-LB\right).\mathrm{rand}+LB\right).\times \partial \end{array}$$

The parameters of the exploitation adjustment are assigned a value of 0.1 in this context. UB and LB are boundary values. In this, we have proposed Eq. 7 for choosing a random number between o and l, which is calculated using a logistics map. The mathematical expression for the random value is given in Eq. 7.

(7) $$\mathrm{rand}=LB+\mathrm{rand}\left(0,1\right)\times \left(UB-LB\right)$$

Subsequently, the arithmetic crossover is performed, in which two regions are randomly selected, and by performing linear combination 2 offspring are produced.

Narrowed Exploitation ( $X_{4}$ ): Executing a strategy involving pursuit and ground-based assault, the Aquila pursues prey, following the trajectory of its escape, culminating in an attack on the ground, as mathematically articulated in Eq. 8–11.

(8) $$\begin{array}{l} X_{4}\left(t+1\right)=QF\times X_{\mathrm{best}}\left(t\right)-\\ \left(G_{1}.X\left(t\right).\mathrm{rand}-G_{2}.LF\left(D\right)+\mathrm{rand}\times G_{1}\right) \end{array}$$

(9) $$QF\left(t\right)=t^{\frac{2\times \mathrm{rand}-1}{{\left(1-T\right)}^{2}}}$$

(10) $$G_{1}=2\times \mathrm{rand}-1$$

(11) $$G_{2}=2\times \left(1-\frac{t}{T}\right)$$

Where a current position is denoted as $X\left(t\right)$ , for search strategy balancing quality function value and is indicated as $QF\left(t\right)$ . During the tracking of prey, Aquila’s movement parameter is denoted by G1. When chasing the prey, the slope of flight is termed as G2, which is minimized linearly from 2 to 0.

Algorithm 1 describes the steps of proposed SIAO algorithms.

##### Algorithm 1: Proposed SIAO

Step 1: Initialization Phase.

Commence by initializing the population of the AO.

Initialize the relevant parameters associated with AO.

WHILE (termination condition) do.

Calculate the values of the fitness function.

$X_{\mathrm{best}}\left(t\right)$ finds the best solution.

for (i = 1,2…, N) do.

Improve the mean value of the present solution.

Improve the x, y, LF (D), G1, G2


If t≤(2/3)×T then

If r and≤0.5 then

Step 2: Expanded exploration ( $X_{1}$ ).

Improve the present solution using Eq. 1.

If Fitness $X_{1}\left(t+1\right)$ < Fitness (X(t)) then

$$X\left(t\right)=\left(X_{1}\left(t+1\right)\right)$$

If Fitness $\left(X_{1}\left(t+1\right)\right)$ < Fitness ( $X_{\mathrm{best}}\left(t\right)$ ) then

$$X_{\mathrm{best}}\left(t\right)=\left(X_{1}\left(t+1\right)\right)$$

Step 3: Narrowed exploration ( $X_{2}$ ).

Improve the present solution using Eq. 3.

If Fitness $X_{2}\left(t+1\right)$ < Fitness (X(t)) then

$$X\left(t\right)=\left(X_{2}\left(t+1\right)\right)$$

If Fitness $\left(X_{2}\left(t+1\right)\right)$ < Fitness ( $X_{\mathrm{best}}\left(t\right)$ ) then

$$X_{\mathrm{best}}\left(t\right)=\left(X_{2}\left(t+1\right)\right)$$

 If thenr and≤0.5

The rand is calculated using the proposed Eq.

“ $\mathrm{rand}=LB+\mathrm{rand}\left(0,1\right)\times \left(UB-LB\right)$ .”

Step 4: Expanded Exploitation ( $X_{3}$ ).

Improve the present solution detailed in Eq. 6.

If Fitness $X_{3}\left(t+1\right)$ < Fitness (X(t)) then

$$X\left(t\right)=\left(X_{3}\left(t+1\right)\right)$$

If Fitness $\left(X_{3}\left(t+1\right)\right)$ < Fitness ( $X_{\mathrm{best}}\left(t\right)$ ) then

$$X_{\mathrm{best}}\left(t\right)=\left(X_{3}\left(t+1\right)\right)$$

Step 4: Narrowed Exploitation ( $X_{4}$ ).

Improve the present solution detailed in Eq. 8.

If Fitness $\left(X_{4}\left(t+1\right)\right)$ < Fitness (X(t)) then

$$X\left(t\right)=\left(X_{4}\left(t+1\right)\right)$$

If Fitness $\left(X_{4}\left(t+1\right)\right)$ < Fitness ( $X_{\mathrm{best}}\left(t\right)$ ) then

$$X_{\mathrm{best}}\left(t\right)=\left(X_{4}\left(t+1\right)\right)$$

return the best solution ( $X_{\mathrm{best}}$ ).

#### 3.1.2 Solution encoding

**Figure 2:**
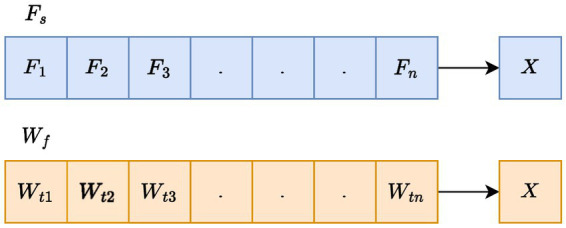
Proposed methodology of CVD prediction.

In this work, the optimization strategy is applied in two phases. For selecting the optimal FS from the extracted feature set F , the selected features are termed as $F_{s}$ . Second, the weight of LSTM indicated as $W_{f}$ is tuned optimally, and the tuned weights are denoted as $W_{f}^{*}$ . The graphical representation in Figure 2 illustrates the input solution for the envisaged SIAO methodology.

### 3.2 Classification process via hybrid LSTM-QNN classifier

**Figure 3:**
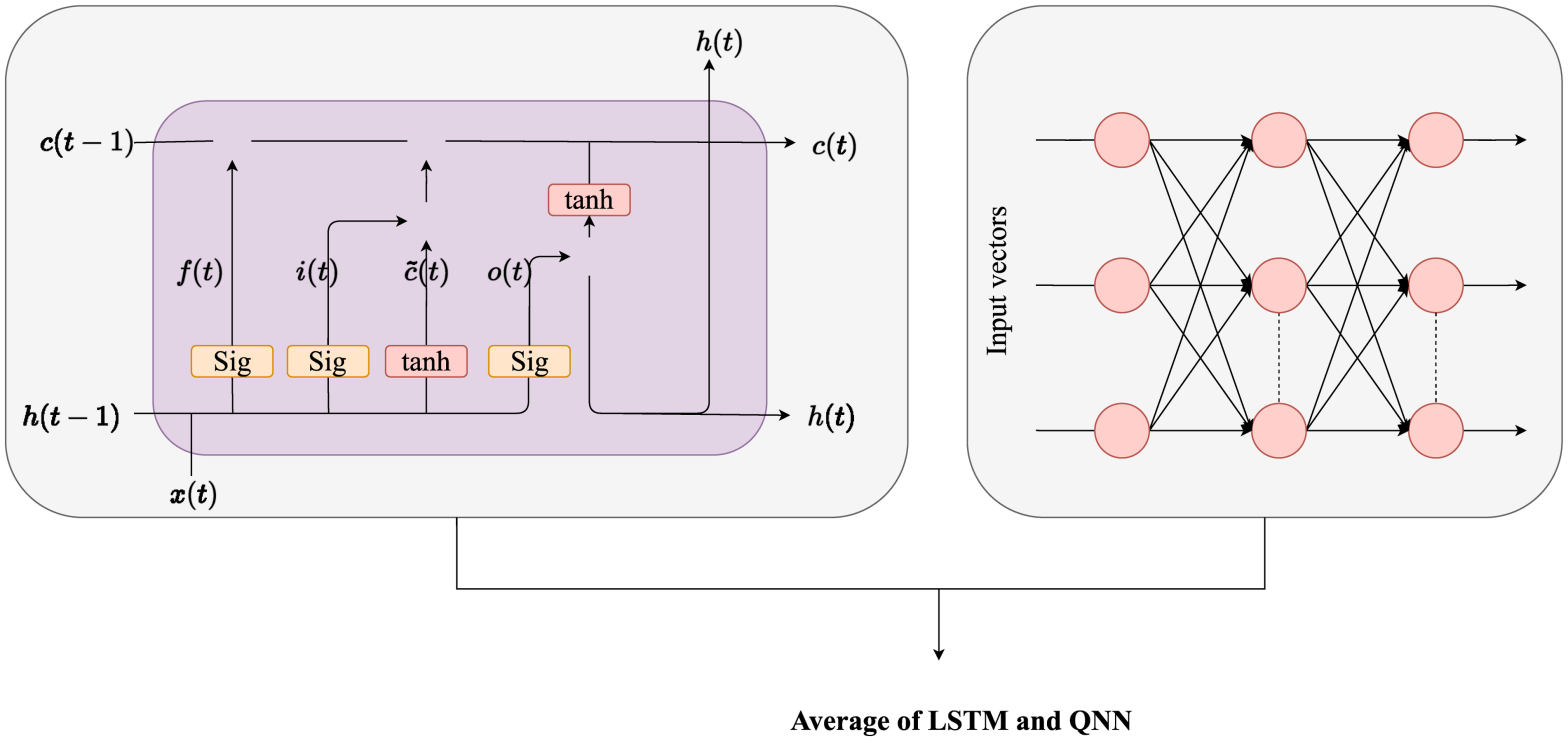
Hybrid model (Average of LSTM and QNN).

As delineated earlier, the optimal features chosen undergo integration into a hybrid classifier for disease presence prediction. To augment the classifier’s performance, the fine-tuning of LSTM weights is intricately executed through the application of the proposed SIAO methodology (Figure 3).

#### 3.2.1 LSTM model

The learning outcome of RNN influences the base theory of LSTM. LSTM can study the lengthy dependencies among variables (21). The long-period series is evaluated using LSTM pseudocode. Activation functions like tanh and sigmoid are essential for NNs, as they introduce non-linearity, allowing the network to tackle complex data patterns and decisions. The resultant outcome enhances the explosion gradient disappearance of the NN algorithm. For controlling the process of memorizing LSTM uses the mechanism called Gating. The unit of LSTM comprises three gates namely input, output, and forget gates.

1. *Forget Gate:* Here, the attention and ignorance of information are decided. Through the function of the sigmoid, the information from the current input and hidden state is passed where the current input is denoted as $X\left(t\right)$ and the hidden state is indicated as $h\left(t-1\right)$ . 0 and 1 are the range of values generated by the sigmoid function. For the point-by-point multiplication, the value of $f\left(t\right)$ is used in Eq. 12.

(12) $$f_{t}=\sigma \left(W_{f.}\left[h_{t-1},x_{t}\right]+b_{t}\right)$$

where timestamp id is denoted as t, $f_{t}$ denotes the forget gate of t, input is determined as $x_{t}$ , $h_{t-1}$ is the previous hidden state, $W_{f}$ signifies weight matrix, and $b_{t}$ denotes the connection bias at timestamp t .

2. *Input Gate:* Here, the operations were performed to update the status of cells. The current position state and hidden position state are projected into the function of the sigmoid. The transformation of values takes place between 0 and 1. Then the same information will get passed to the function of the. For performing network regulation, the tanh operator generates a value range between 0 and 1. The generated values are ready for point-by-point multiplication in Eq. 13–17.

(13) $$i_{t}=\sigma \left(W_{i}.\left[h_{t-1},x_{t}\right]+b_{i}\right)$$

(14) $${\tilde{C}}_{t}=\tanh\left(W_{c}.\left[h_{t-1},x_{t}\right]+b_{c}\right)$$

Where, $W_{i}$ denotes the weight matrix, $b_{i}$ indicated the bias vector at t, the value generated by tanh is denoted as ${\tilde{C}}_{t}$ , weight matrix of the tanh operator between cell state information and network output is indicated as $W_{c}$ , and the bias vector is represented as $b_{c}$ .

3. *Cell state:* The subsequent step is to select and save the information in the cell state. The multiplication is performed for the previous cell state and forgets the vector. If the value of the resultant outcome is 0, then in the cell state the value will drop. Then the point-by-point addition is performed by the output value of the vector in the input.

(15) $$C_{t}=f_{t}\times C_{t-1}+i_{t}\times {\tilde{C}}_{t}$$

Here, the cell state of information is denoted as $C_{t}$ , the previous timestamp is indicated by $C_{t-1}$ , and the value generated by tanh is expressed as ${\tilde{C}}_{t}\text{.}$

4. *Output Gate:* To determine the value of the hidden state, the output gate is utilized. In this state, the information on the inputs that came before it is stored. Within the beginning, the sigmoid function will be given both the value of the current state as well as the value of the hidden state that came before it. A new cell state will be generated as a result of this, and it will be sent to the function that is responsible for calculating tanh. After that, a multiplication operation will be carried out on those outputs on a point-by-point basis. The network decides the information that is carried out for the hidden state based on the results that it has obtained. The hidden state that is produced as a result is then utilized for prediction.

(16) $$o_{t}=\sigma \left(W_{o}.\left[h_{t-1},x_{t}\right]+b_{o}\right)$$

(17) $$h_{t}=o_{t}\times \tanh\left(C_{t}\right)$$

Where the output gate at t is denoted by $o_{t}$ , out gates’ weight matrix is indicated by $W_{o}$ , a vector is represented as $b_{o}$ , and the output of LSTM is mentioned as $h_{t}$ .

#### 3.2.2 QNN model

A QNN (22), as elucidated in reference, constitutes a multi-layered feedforward NN renowned for its efficacy in classifying uncertain data. The QNN state shift function embodies a linear composition of multiple sigmoid functions, commonly referred to as a multi-level switch function. Unlike the binary expression of traditional sigmoid functions with two states, the QNN’s hidden layer neural cells exhibit a richer spectrum of states. Introducing a discrete quantum interval for the sigmoid function allows for the mapping of diverse data onto distinct levels, affording enhanced classification flexibility. The quantum interval within a QNN is acquired through a training process. Structurally, a QNN comprises input, hidden, and output layers, with the output function of the hidden layer mathematically articulated in Eq. 18.

(18) $$b_{r}=\frac{1}{\mathrm{ns}}\overset{\mathrm{ns}}{\underset{s=1}{\sum }}f\left[\beta \left(W^{T}X-\theta_{s}\right)\right]$$

Where, γ=1,2,3,…u and $f\left(x\right)=1/1\left(1+\exp\left(-x\right)\right)$ is an excitation function in which W is expressed as the weight of the network, X is the input vector, the slope factor is indicated as β , the input of the quantum cell is represented as $W^{T}X$ , and the quantum interval is termed as $\theta_{s}\text{.}$

### 3.3 Preprocessing phase

The preprocessing phase is conducted as an initial step to assess the data quality. Data cleaning is performed to eliminate incorrect and incomplete data. Additionally, null values and duplicate entries are removed during this preprocessing phase.

#### 3.3.1 Central tendency

Toward a central point the size of the sample inclined toward infinity. This data property is termed a central tendency and the point toward the data gets inclined is termed a central tendency measure (23). A central propensity can be suitable for both a constrained association of features and for a theoretical transference. Moreover, some of the measures of central tendency for n data points with value $I_{\mathrm{data}\left(i\right)}$ extracted in our proposed model are given as follows:

1. *Arithmetic Mean* (*AM,* $\bar{I_{\mathrm{data}\left(i\right)}}$ ): The arithmetic mean, a fundamental measure of central tendency, is denoted as the sum of all data annotations divided by the total number of data points. Eq. 19 expresses the mean of the data.

(19) $$\bar{I_{\mathrm{data}\left(i\right)}}=\frac{1}{n}\overset{n}{\underset{i=1}{\sum }}I_{\mathrm{data}\left(i\right)}$$

2. *Median:* A statistical metric denoting the central value within a dataset, effectuates a division of the dataset into two equidistant halves. This partition is achieved through the meticulous arrangement of data points in ascending order, facilitating the identification of a singular data point characterized by an equitable distribution of values both superior and inferior to it. The methodology for ascertaining the median subtly diverges contingent on whether the dataset harbors an odd or even count of values. Eq. 20 elucidates the mathematical formulation encapsulating the concept of the median.

(20) $$\mathrm{Medi}an=\left[\frac{I_{\mathrm{data}\left(i\right)}+1}{2}\right]$$

3. *Mode:* In the dataset, one of the frequently occurring values is the mode. The mode is also a degree of central tendency that identifies the group or rating that happens the maximum often inside the distribution of data.
4. *Standard deviation* (*SD,* σ ): In statistics, standard deviation measures the dataset dispersion relative to the mean. Also, the SD is calculated as the variance square root. Eq. 21 denotes the mathematical expression for SD.

(21) $$\sigma =\sqrt{\frac{1}{n}}\overset{n}{\underset{i=1}{\sum }}{\left(I_{\mathrm{data}\left(i\right)}-\bar{I_{\mathrm{data}\left(i\right)}}\right)}^{n}$$

The minimum value obtained was considered as the initial order statistics and the maximum value is the last order statistics.

5. *Geometric mean (GM)*: A sophisticated measure of central tendency, that computes the product of specified values in a numerical series. Importantly, it is undefined if any element in the series is negative or zero, as succinctly expressed in Eq. 22.

(22) $$GM={\left(\overset{n}{\underset{i=1}{\prod }}I_{\mathrm{data}\left(i\right)}\right)}^{\frac{1}{n}}$$

6. *Harmonic Mean (HM)*: Delineated as the reciprocal of the AM, computed from the reciprocals of individual annotations. Its evaluation is confined to a comprehensive "positive scale," ensuring meticulous consideration of positive values exclusively. Eq. 23 elegantly captures the intricate mathematical formulation underpinning the HM.

(23) $$HM=\frac{n}{\sum_{i=1}^{n}\left(\frac{1}{I_{\mathrm{data}\left(i\right)}}\right)}$$

7. *Trimmed Mean (TM):* It encompasses the determination of the mean following the selective omission of specific elements from the extremes of a probability distribution or pattern. This procedure uniformly excludes an equal quantity from both the high and low ends.
8. *Interquartile range (IQR):* Within statistical analysis, IQR assumes a pivotal role as a metric to gauge the dispersion of data and observations. The precise mathematical notation for IQR is succinctly expressed in Eq. 24, providing an exact quantification of this statistical characteristic.

(24) $$IQR=\frac{2}{n}\overset{\frac{3.n}{4}}{\underset{i=\frac{n}{4}+1}{\sum }}I_{\mathrm{data}\left(i\right)}$$

9. *Midrange:* The midrange is defined as the mean of the maximum and minimum number in the dataset. It is expressed mathematically in Eq. 25.

(25) $$M_{rg}=\frac{\mathrm{low}\left(I_{data\left(i\right)}\right)+\mathrm{high}\left(I_{\mathrm{data}\left(i\right)}\right)}{2}$$

10. *Midhinge:* The midhinge is considered as the estimation of central tendency (C) shown in Eq. 29.

(26) $$M_{\mathrm{hg}}=\bar{C_{1,3}\left(I_{\mathrm{data}\left(i\right)}\right)}=\frac{C_{1}\left(I_{\mathrm{data}\left(i\right)}\right)+C_{3}\left(I_{\mathrm{data}\left(i\right)}\right)}{2}$$

11. *Trimean:* A trimean is represented as the general tendency of a data set and its mathematical notation is given in Eq. 27 where $C_{1}$ , $C_{2}$ , $C_{3}$ are central tendencies for quartiles.

(27) $$Tri_{m}=\frac{C_{1}+2.C_{2+}C_{3}}{4}$$

12. *Winsorized means:* This method pertains to an averaging technique that initially substitutes the smallest and largest values with the annotations nearest to them. This strategic replacement is executed to mitigate the influence of anomalous extreme values during the computation process.

#### 3.3.2 Degree of dispersion

In statistical analysis, dispersion, also interchangeably referred to as variability, spread, or scatter, characterizes the degree of deviation or spreading inherent within a distribution (24). This metric delineates the extent to which data points diverge or converge from a central tendency, offering valuable insights into the distribution’s inherent dynamics.

1. *IQR:* Serves as a sophisticated metric embodying statistical dispersion, elucidating the nuanced spread encapsulated between the 75 and 25 percentiles. This measure offers a granular depiction of variability by meticulously assessing the interquartile span.
2. *Range:* In the domain of statistical analysis, the Range assumes the role of a fundamental measurement, meticulously quantifying the explicit divergence existing between the uppermost and lowermost values within a dataset. This metric provides an unambiguous reflection of the dataset’s inherent variability.
3. *Mean absolute difference (MAD):* It is a quantitative facet of dispersion, that delineates the dissonance between two independently drawn values from a probability distribution. This metric affords a nuanced insight into the distributional nuances characterizing the dataset.
4. *Average absolute deviation (AAD):* It assumes the mantle of quantifying the normative divergence of data points from the pivotal central tendency within an informational index, thereby encapsulating the comprehensive variability inherent in the dataset.
5. *Distance standard deviation:* In the insight’s hypothesis, the departure distance relationship is a fraction of dependence between two mutually uneven vectors of unrestricted measurement. A diverse fraction of divergence is “dimensionless.”
6. *Coefficient of* Var*iation (CV):* It ensconced within the domain of probability statistics, and surfaces as a comprehensive barometer of dispersal within a probability or recurrence distribution. Articulated as a ratio, the CV serves as a standardized gauge, representing the fraction of SD to the mean.
7. *Quartile coefficient of dispersion (QCD):* A nuanced statistical metric, that assumes symbolic relevance in evaluating divergence within a dataset. Its precise calculation leverages the first ( $P_{1})$ and third ( $P_{3}$ ) quartiles for each dataset, culminating in the articulation of the scattering coefficient, as rigorously expressed in Eq. 28.

(28) $$QCD=\frac{P_{3}-P_{1}}{P_{3}+P_{1}}$$

8. *Replicating the coefficient of Gini & relative mean difference:* MAD, which is a precise measure of accurate divergence equivalent to the AAD of 2 independent attributes drawn from a probability distribution. A noteworthy metric associated with MAD is the AAD, representing the MAD divided by the AM and twice the Gini coefficient.
9. *Entropy (H):* The entropy of a discrete variable displays invariance in both location and scale, signifying inherent scale independence. In contrast to traditional dispersion measures, the entropy of a continuous variable remains constant across regions and seamlessly accommodates new information, exhibiting a unique scalability. The entropy function $H\left(y\right)$ for continuous variable x , c can be arithmetically expressed in Eq. 29.

(29) $$H\left(y\right)=H\left(x\right)+\log\left(c\right)$$

#### 3.3.3 Qualitative variation (QV)

This index is the *measure of arithmetical dispersion in the ostensible distribution* (25). Between the 0 and 1 bounds, the data normalization exists and then changes to level 4. The data level changes are expressed in Table 2.

**Table 2:** Transformed data levels.

Datapoint transferred	Data range normalized
1	If 0–0.25
2	If 0.25–0.5
3	If 0.5–0.75
4	If 0.75–1

Twenty-three features are there in the QV index. Also, indices of Wilcox’s and its characteristics include RanVR, MNDif, R packages, ModVR, B index, HRel, StDev, MNDif, and AvDev. Gibbs’ indices include M1, M2, M4, and M6, while single-order sample indices encompass Menhinick’s, Lloyd & Ghelardi’s, Shannon–Wiener, Average taxonomic distinctness, Hill’s diversity numbers, Theil’s H, Brillouin, McIntosh’s D and E, Cotgreave’s, Bulla’s E, Berger–Parker, Index of qualitative variation, Margalef’s, Caswell’s V, Rarefaction, Smith and Wilson’s B, Q statistic, Harvey, Camargo’s, E, Smith & Wilson’s, Simpson’s, Heip’s, Rényi entropy, Strong’s, Horn’s, and Fisher’s alpha. $F_{QV}$ determined the characteristics of extracted qualitative variation.

#### 3.3.4 Symmetric uncertainty

The characteristics and class of symmetric uncertainty are evaluated based on the estimated SU relationship metric (26). The communal information is calculated using Eq. 30.

(30) $$CM\left(Q,P\right)=\sum PO\left(Q,B\right)\log_{2}\frac{PO\left(Q,P\right)}{PO\left(Q\right).PO\left(P\right)}$$

In Eq. 31, communal information is indicated by CM , the feature is represented as Q , the class is denied as P , and the function of probability is represented as PO . Also, Eq. 31 indicates symmetrical uncertainty.

(31) $$SU\left(Q,P\right)=2\left(CM\left(Q,P\right)\right)/\left(H\left(Q\right).H\left(P\right)\right)$$

In Eq. 32, the entropy function is indicated as *H*. $F_{SU}$ denotes the symmetric uncertainty feature. So, the entire feature *F* combines the features that are extracted like central tendency $F_{CT}$ , degree of dispersion $F_{D}$ , qualitative variation $F_{QV}$ , and symmetrical uncertainty $F_{SU}$ were termed in Eq. 32.

(32) $$F=F_{CT}+F_{D}+F_{QV}+F_{SU}$$

## 4 Results and discussions

### 4.1 Simulation details

The execution of the CVD prediction model within the Python 3.11 environment involves a systematic evaluation, methodically assessing a plethora of Type I metrics and Type II metrics. This comprehensive scrutiny unfolds across two distinct datasets: Dataset 1, sourced from the Cleveland dataset (UCI Machine Learning Repository, n.d.-a) featuring 76 attributes, with a focused exploration of a refined subset of 14 attributes, notably emphasizing the Cleveland dataset. Meanwhile, Dataset 2, attained from the (UCI Machine Learning Repository, n.d.-b) comprises 13 attributes and an intricately defined cost matrix denoted as ‘abse’ and ‘pres.’ The orchestrated training and testing processes systematically unfold across varied proportions (60, 70, 80, and 90%), providing a structured lens for a nuanced examination of the predictive model’s performance.

$$\begin{array}{cc} \mathrm{absence}\;0 & 1\\ \mathrm{presence}\;5 & 0 \end{array}$$

In the above matrix, the row indicates the true values and the columns predicted.

### 4.2 Performance analysis of the adopted and traditional model for Dataset-1

**Figure 4:**
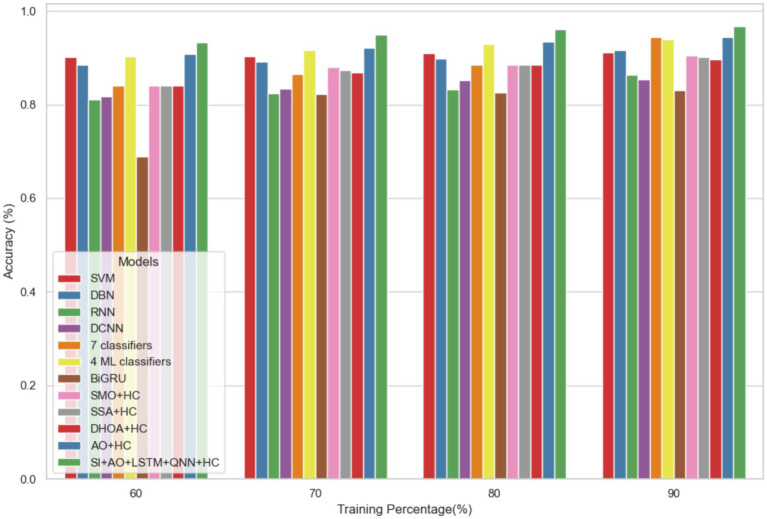
Comparative analysis of the accuracy rates in predicting CVD on Dataset-1.

**Figure 5:**
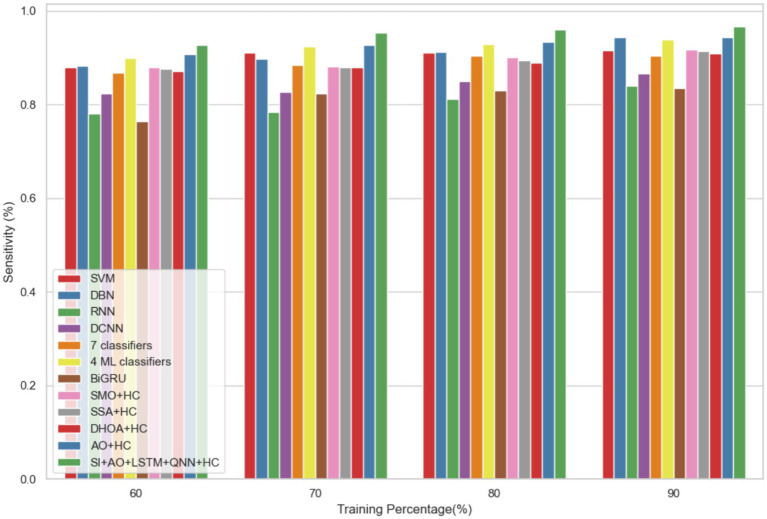
Comparative analysis of the sensitivity rates in predicting CVD on Dataset-1.

**Figure 6:**
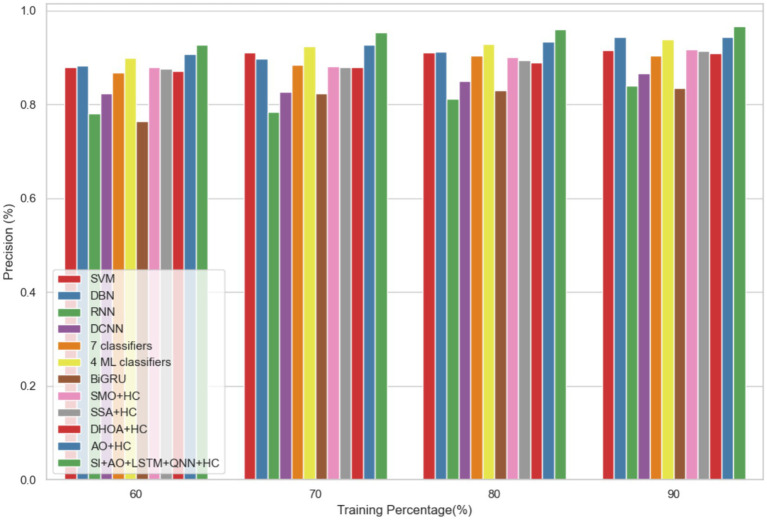
Comparative analysis of the precision rates in predicting CVD on Dataset-1.

The performance of the proposed model is evaluated over the existing models like SVM (21), DBN (Deep Belief Network) (22), RNN (27), DCNN (Deep CNN) (28), 7 classifiers (DT, NB, LR, SVM, k-NN, ANN and Vote (a hybrid technique with NB and LR)) (4), 4 ML classifiers (DT, LR, XGBoost, SVM) (29), BiGRU (Bidirectional Gated Recurrent Unit) (30), SMO (Sequential Minimal Optimization) + HC (Hybrid Classifiers) (26), SSA (Salp Swarm Algorithm) + HC (31), DHOA (Dear Hunting Optimization Algorithm) + HC (32), AO + HC (7), SI + AO + LSTM + QNN + HC, accordingly. The predictive model’s performance is rigorously evaluated through key metrics, including accuracy, precision, and sensitivity, across various learning percentages (60, 70, 80, and 90%). Figure 4 illustrates the exceptional accuracy of the compositional model, achieving a remarkable 95.54% during the 90% training phase. The projected approach consistently surpasses the performance of other existing models at all learning percentages, such as SVM, DBN, RNN, DCNN, 7 classifiers, 4 ML classifiers, BiGRU, SMO + HC, SSA + HC, DHOA + HC, AO + HC, SI + AO + LSTM + QNN + HC. Figure 5 sheds light on the superior sensitivity of the proposed SI-AO-LSTM-QNN approach, particularly evident with a peak sensitivity of 95.86% at the 90% training percentage. This notable performance outshines other existing approaches. Sensitivity rates for the 60, 70, and 80% training percentages are also substantial, standing at 91.6, 92.95, and 94.39%, respectively. Precision analysis, as depicted in Figure 6, further emphasizes the prowess of the proposed model. Achieving the highest precision rate of 96.03% during the 90% training phase, the SI-AO-LSTM-QNN approach outperforms the already existing models. Precision rates for the other training percentages are commendable, measuring at 92.76, 94.33, and 95.47%.

Table 3 provides a comprehensive performance analysis for the prediction of CVD on Dataset 1, focusing on a Training percentage (TP) of 90%. Various metrics, including accuracy, sensitivity, specificity, precision, recall, F1-score, Matthews Correlation Coefficient (MCC), Negative Predictive Value (NPV), False Positive Rate (FPR), and False Negative Rate (FNR), are reported for a range of existing models, as well as the proposed model, SI + AO + LSTM + QNN + HC. Notably, the proposed model achieves outstanding results with an accuracy of 96.69%, sensitivity of 96.62%, specificity of 96.77%, precision of 96.03%, recall of 97.86%, F1-score of 96.85%, MCC of 96.37%, NPV of 96.25%, FPR of 3.23%, and FNR of 3.38%. These metrics collectively indicate the superior predictive capabilities of the proposed SI + AO + LSTM + QNN + HC model, showcasing its robust performance compared to other existing models across a diverse set of evaluation criteria.

**Table 3:** Performance analysis for prediction of CVD of dataset 1 for TP = 90%.

Metrics	Accuracy	Sensitivity	Specificity	Precision	F1-score	MCC	NPV	FPR	FNR
SVM (33)	0.91079	0.915957	0.91079	0.91079	0.91079	0.91079	0.914113	0.08921	0.084043
DBN (34)	0.916754	0.943232	0.921099	0.923449	0.918028	0.91879	0.939337	0.078901	0.056768
RNN (29)	0.863295	0.83929	0.884078	0.864878	0.851892	0.780951	0.861998	0.115922	0.16071
DCNN (6)	0.854458	0.865934	0.852423	0.840533	0.853045	0.724256	0.876004	0.147577	0.134066
7 classifiers (4)	0.944006	0.904322	0.944193	0.94492	0.944902	0.877832	0.90148	0.055807	0.095678
4 ML classifier (9)	0.939135	0.938981	0.939321	0.940045	0.940026	0.928548	0.936801	0.060679	0.061019
BiGRU (25)	0.831094	0.834353	0.836954	0.803626	0.818701	0.704706	0.862893	0.163046	0.165647
SMO + HC (35)	0.905631	0.917888	0.891647	0.899332	0.908415	0.871494	0.913507	0.108353	0.082112
SSA + HC (30)	0.901581	0.91371	0.887748	0.895251	0.904286	0.868333	0.909493	0.112252	0.08629
DHOA + HC (28)	0.896831	0.908896	0.883071	0.890535	0.899522	0.863758	0.904701	0.116929	0.091104
AO + HC (7)	0.94413	0.943851	0.945169	0.948321	0.94577	0.933364	0.94166	0.054831	0.056149
LSTM (32)	0.828358	0.735632	0.961921	0.965309	0.834964	0.689585	0.7164	0.038079	0.264368
QNN (21)	0.9273	0.9461	0.9075	0.9136	0.9295	0.8740	0.9428	0.0924	0.0538
Proposed model	0.966922	0.966244	0.967714	0.9603	0.968473	0.963715	0.962534	0.032286	0.033756

### 4.3 Performance analysis of the adopted and traditional model for Dataset-2

**Figure 7:**
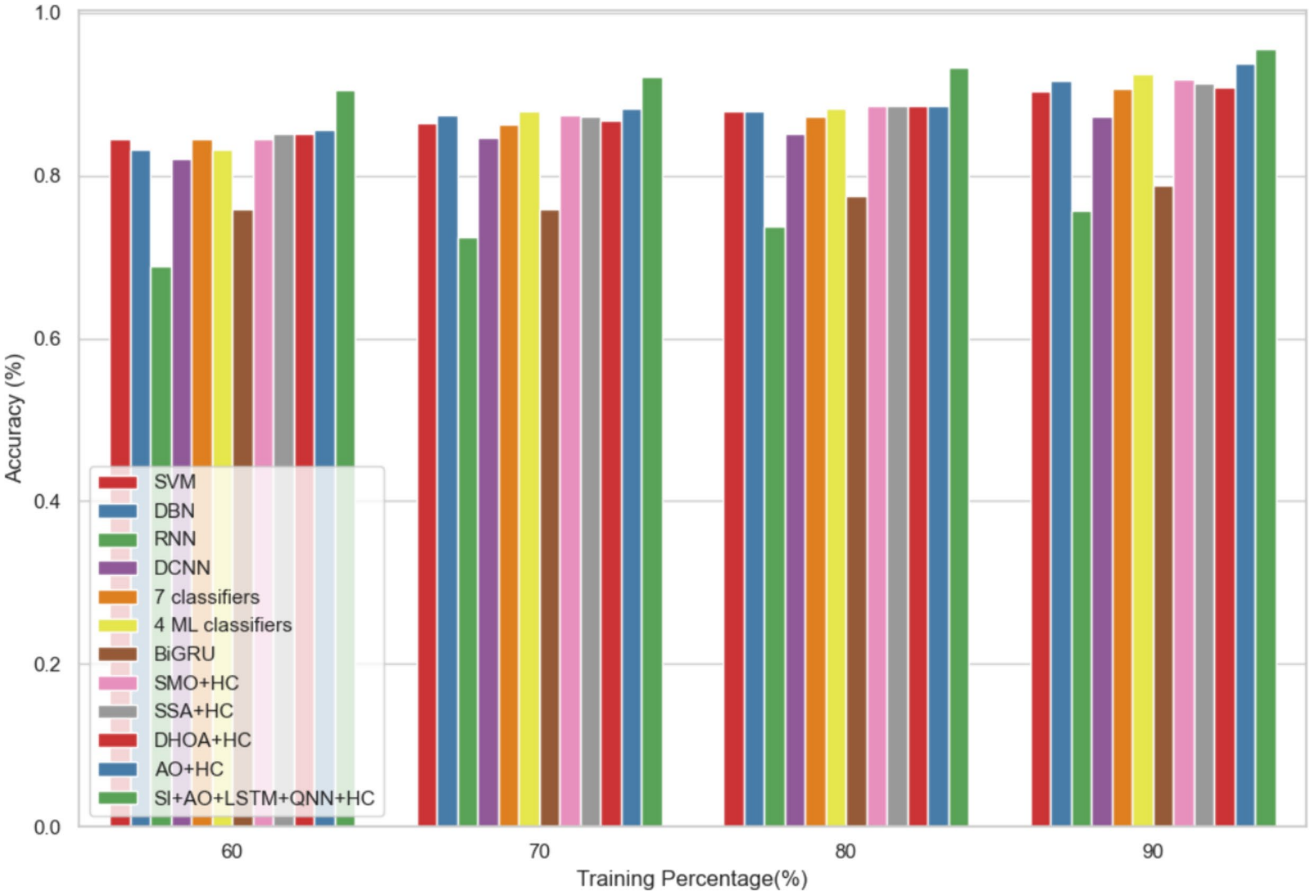
Comparative analysis of the accuracy rates in predicting CVD on Dataset-2.

**Figure 8:**
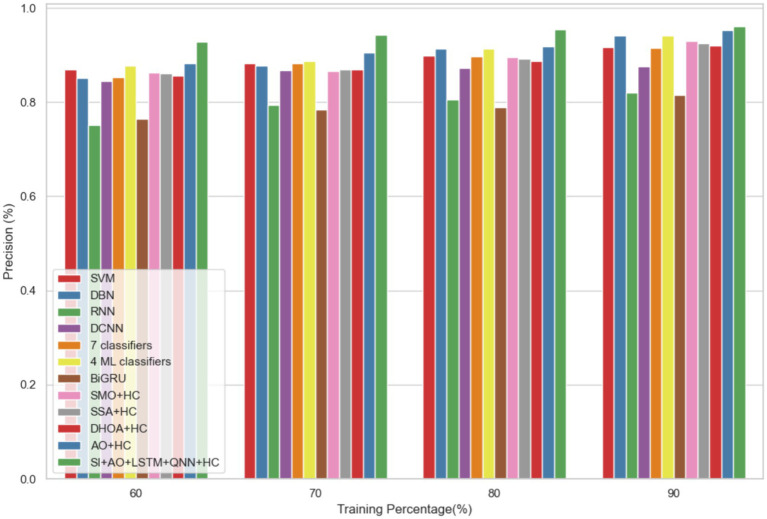
Comparative analysis of the precision rates in predicting CVD on Dataset-2.

**Figure 9:**
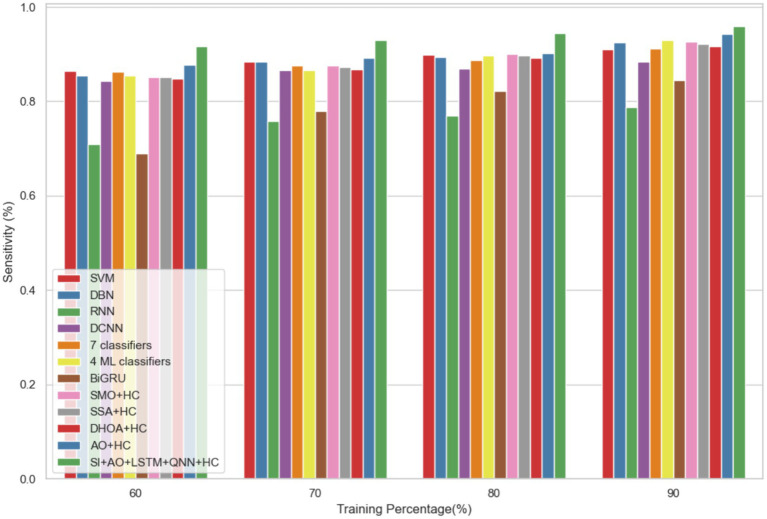
Comparative analysis of the sensitivity rates in predicting CVD on Dataset-2.

In dataset 2, the proposed model is compared to SVM, DBN, RNN, DCNN, 7 classifiers, 4 ML classifiers, BiGRU, SMO + HC, SSA + HC, DHOA + HC, AO + HC, SI + AO + LSTM + QNN + HC, and others. Notably, the SI-AO-LSTM-QNN approach consistently outperforms the existing models, achieving higher values across critical metrics. Specifically, for accuracy, sensitivity, and precision, the proposed model attains impressive rates of 96.69, 96.62, and 96.03%, respectively. These superior metrics are observed consistently across various learning percentages, with the highest values achieved at the 90th learning percentage. Figure 7 visually represents the accuracy comparison, illustrating that the SI-AO-LSTM-QNN model excels, achieving the highest accuracy among the compared models. Figure 8 showcases the precision performance, indicating higher values, especially at the 80th and 90th learning percentages. Lastly, Figure 9 presents the sensitivity analysis, highlighting the consistently superior sensitivity of the proposed model across different training percentages.

Table 4 provides a comprehensive performance analysis for the prediction of CVD on Dataset 2, with a focus on the TP rate of 90%. Various metrics, including accuracy, sensitivity, specificity, precision, F1-score, MCC, NPV, FPR, and FNR, are reported for a range of existing models, as well as the proposed model SI + AO + LSTM + QNN + HC. The SI + AO + LSTM + QNN + HC model outshines the other models consistently across all metrics, achieving an accuracy of 95.55%, sensitivity of 95.87%, specificity of 94.52%, precision of 96.03%, F1-score of 96.94%, MCC of 93.09%, NPV of 94.67%, FPR of 5.48%, and FNR of 4.13%. These superior metrics signify the robust predictive capabilities of the proposed model, showcasing its effectiveness in comparison to a diverse set of existing models across a spectrum of evaluation criteria on Dataset-2.

**Table 4:** Performance analysis for prediction of CVD of Dataset 2 for TP = 90%.

Metrics	Accuracy	Sensitivity	Specificity	Precision	F1-score	MCC	NPV	FPR	FNR
SVM (33)	0.903943	0.91079	0.884254	0.916544	0.918485	0.887562	0.898065	0.115746	0.08921
DBN (34)	0.91717	0.925456	0.89554	0.941486	0.937214	0.867554	0.885457	0.10446	0.074544
RNN (29)	0.757692	0.788	0.774623	0.820833	0.804082	0.754632	0.779862	0.225377	0.212
DCNN (6)	0.873491	0.884144	0.812193	0.875556	0.880953	0.782849	0.841727	0.187807	0.115856
7 classifiers (4)	0.907628	0.911215	0.892074	0.914643	0.9168	0.884667	0.905379	0.107926	0.088785
4 ML classifier (9)	0.925602	0.929508	0.912176	0.941193	0.939203	0.870667	0.910303	0.087824	0.070492
BiGRU (25)	0.788	0.844286	0.745249	0.815172	0.829474	0.780115	0.758131	0.254751	0.155714
SMO + HC (35)	0.918383	0.925771	0.89847	0.92913	0.931322	0.884695	0.912411	0.10153	0.074229
SSA + HC (30)	0.913569	0.920919	0.893732	0.924301	0.926481	0.880019	0.907629	0.106268	0.079081
DHOA + HC (28)	0.908756	0.916067	0.888993	0.919472	0.921641	0.875343	0.902847	0.111007	0.083933
AO + HC (7)	0.938558	0.943341	0.921635	0.952722	0.952239	0.888595	0.916232	0.078365	0.056659
LSTM (32)	0.8703	0.8703	0.8703	0.9306	0.8995	0.7206	0.7704	0.1296	0.1296
QNN (21)	0.9079	0.9328	0.862	0.9258	0.9293	0.7973	0.874	0.1379	0.0671
Proposed model	0.955479	0.958691	0.945167	0.9603	0.969417	0.930939	0.946673	0.054833	0.041309

### 4.4 Convergence analysis

**Figure 10:**
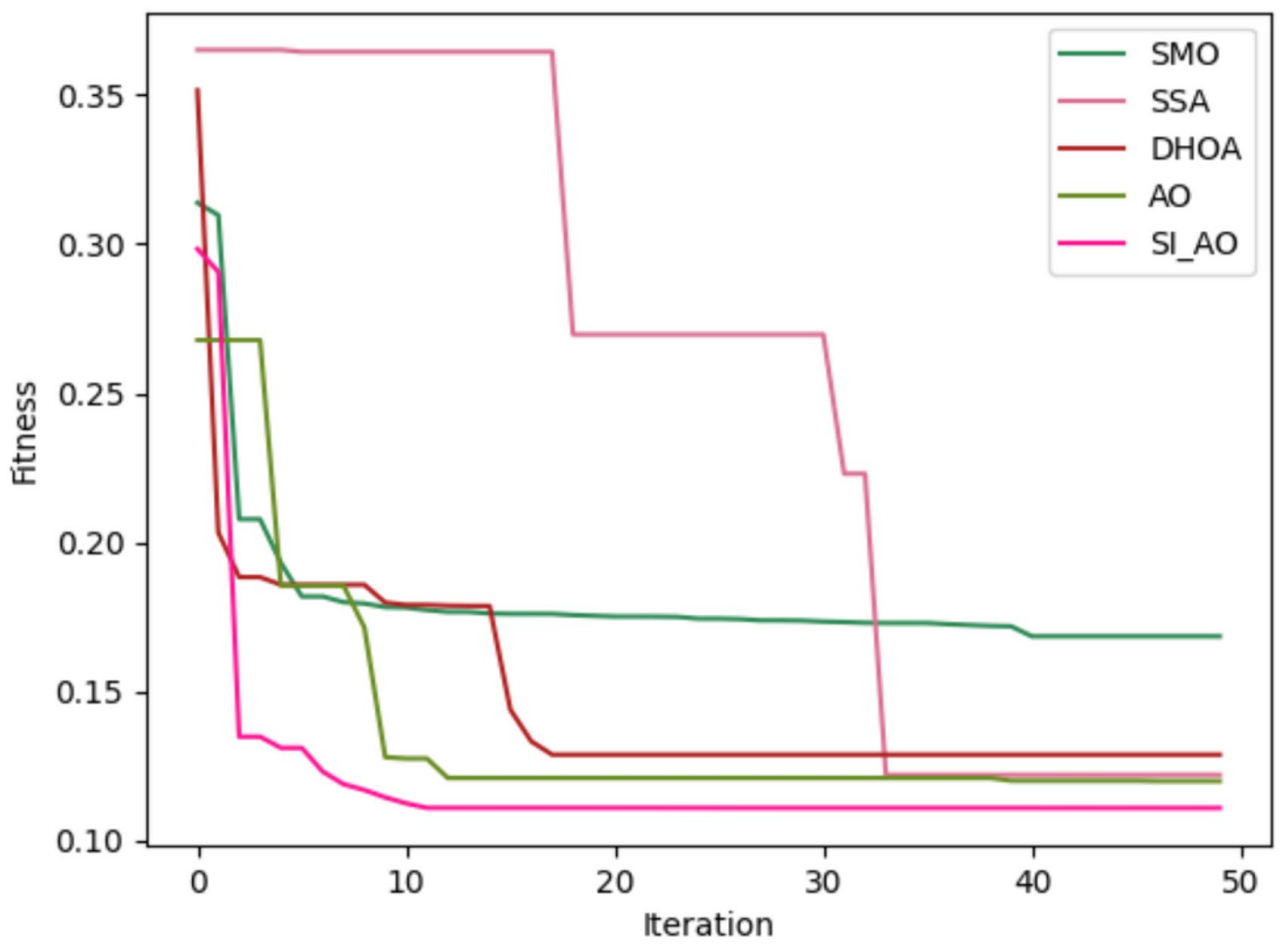
Convergence analysis for Dataset-1.

**Figure 11:**
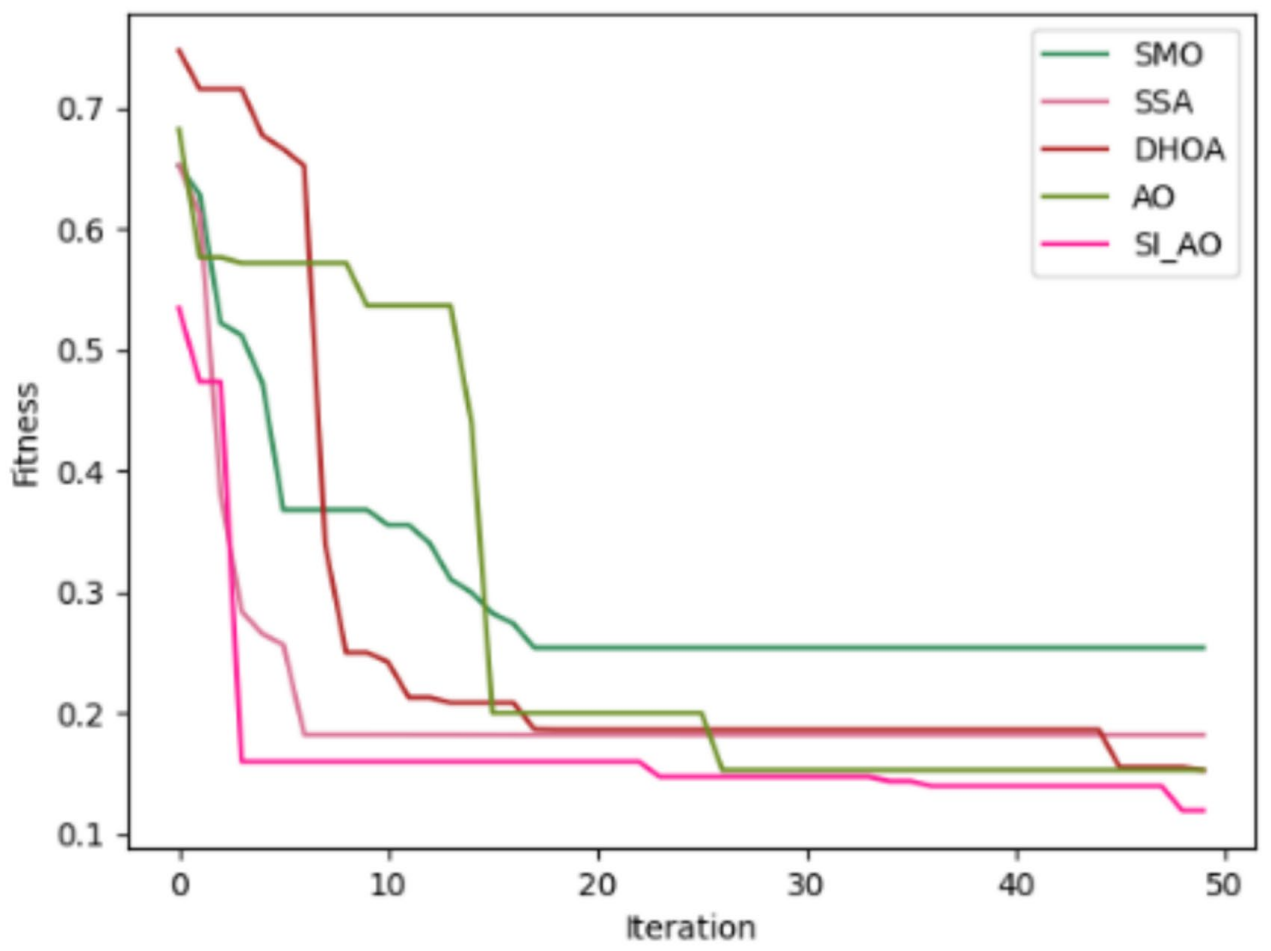
Convergence analysis for Dataset-2.

Convergence analysis of the proposed SI-AO-LSTM-QNN, in comparison to conventional methods like SMO, SSA, DHOA, AO, and SI-AO, is visually presented in Figures 10, 11. The primary objective of the adopted methodology revolves around convergence analysis, with a specific focus on maximizing accuracy. The analysis reveals that heightened convergence is achieved with an increase in the iteration count. Given the inverse relationship between accuracy and errors, the overarching goal of this research is to attain the highest possible detection accuracy, thereby minimizing error rates. In Figure 10, which pertains to Dataset-1, the graphical representation illustrates superior convergence of the proposed work over existing counterparts, with maximal convergence evident at the 20th iteration. Likewise, in Figure 11, corresponding to Dataset-2, the presented work demonstrates robust convergence, surpassing other methods and reinforcing its effectiveness in the classification.

### 4.5 Statistical analysis

Tables 5, 6 provide a comparative statistical analysis of accuracy for the proposed SI-AO-LSTM-QNN model against traditional schemes on Dataset-1 and Dataset-2, respectively. The stochastic nature of the optimization algorithm led to five independent runs, and statistical measures such as mean, SD, median, worst, and best were recorded for accuracy. In Table 6 for Dataset-1, the proposed SI-AO-LSTM-QNN model showcases a superior mean performance of 95.23%, outperforming traditional methods. Notably, the method exhibits a narrow SD of 1.279, indicating consistency across runs. The worst-case scenario is observed at 93.31%, and the best-case scenario attains an impressive 96.69%. In comparison, other traditional methods show varying performance levels, with SI-AO-LSTM-QNN consistently demonstrating higher accuracy.

**Table 5:** Comparative statistical analysis of accuracy for proposed and traditional schemes in Dataset-1.

Method	Mean	Median	SD	Worst	Best
SVM (33)	90.64	90.65	0.367	90.18	91.07
DBN (34)	89.81	89.50	1.156	88.58	91.67
RNN (29)	83.27	82.81	1.914	81.11	86.32
DCNN (6)	83.92	84.28	1.505	81.67	85.44
7 classifiers (4)	88.35	87.49	3.830	84.03	94.40
4 ML classifier (9)	92.25	92.35	1.337	90.37	93.91
BiGRU (25)	79.21	82.39	5.930	68.95	83.10
SMO + HC (35)	87.79	88.25	2.334	84.10	90.56
SSA + HC (30)	87.52	87.95	2.243	84.03	90.15
DHOA + HC (28)	87.29	87.72	2.119	84.03	89.68
AO + HC (7)	92.73	92.83	1.348	90.83	94.41
LSTM (32)	95.23	95.45	1.279	93.31	96.69

**Table 6:** Comparative statistical analysis of accuracy for proposed and traditional schemes in Dataset-2.

Method	Mean	Median	SD	Worst	Best
SVM (33)	87.33	87.24	2.15	84.45	90.39
DBN (34)	87.62	87.76	3.00	83.25	91.71
RNN (29)	72.73	73.10	2.48	68.95	75.76
DCNN (6)	84.82	84.91	1.85	82.13	87.34
7 classifiers (4)	87.21	86.81	2.28	84.48	90.76
4 ML classifier (9)	88.00	88.11	3.29	83.25	92.56
BiGRU (25)	77.02	76.69	1.19	75.92	78.80
SMO + HC (35)	88.09	88.02	2.62	84.48	91.83
SSA + HC (30)	88.07	87.91	2.25	85.13	91.35
DHOA + HC (28)	87.84	87.68	2.13	85.13	90.87
AO + HC (7)	89.10	88.45	2.97	85.65	93.85
LSTM (32)	92.90	92.74	1.81	90.56	95.54

### 4.6 Analysis on features

Tables 7, 8 provide an in-depth analysis of feature performance in predicting CVD for Dataset-1 and Dataset-2, respectively. In Dataset-1, the proposed feature exhibits superior predictive capabilities with an accuracy of 95.59%, outperforming scenarios without FS (94.58%) and optimization (94.62%). The proposed feature also excels in key metrics such as F1-score, precision, sensitivity, specificity, MCC, NPV, FPR, and FNR, underscoring its effectiveness in enhancing the overall predictive accuracy for CVD in Dataset-1. Specifically, the proposed feature demonstrates improved sensitivity and NPV, suggesting its robust ability to correctly identify positive cases and avoid false negatives.

**Table 7:** Feature analysis for Dataset-1.

Metrics	Without FS	Without optimization	Proposed feature
Accuracy	0.945848375	0.946277097	0.955888608
F1-score	0.947162427	0.959159303	0.963933803
Precision	0.952755906	0.96353167	0.958619611
Sensitivity	0.941634241	0.954826438	0.969368891
Specificity	0.950331126	0.92962963	0.930392593
MCC	0.891698291	0.880765057	0.915424716
NPV	0.938675388	0.91355778	0.950517634
FPR	0.049668874	0.07037037	0.06960741
FNR	0.058365759	0.045173562	0.03063111

**Table 8:** Feature analysis for Dataset-2.

Metrics	Without FS	Without optimization	Proposed feature
Accuracy	0.934916	0.943396	0.96652
F1-score	0.939274	0.956938	0.974655
Precision	0.922979	0.961538	0.969282
Specificity	0.911591	0.925926	0.940741
Sensitivity	0.956864	0.952381	0.980151
MCC	0.870635	0.87446	0.925606
NPV	0.949849	0.909091	0.96109
FPR	0.088409	0.074074	0.059259
FNR	0.043136	0.047619	0.019849

Turning attention to Dataset-2 in Table 8, the proposed feature showcases exceptional predictive performance, achieving an accuracy of 96.65% compared to scenarios without FS (93.49%) and optimization (94.34%). The proposed feature consistently outperforms across various metrics, emphasizing its importance in accurate CVD prediction. Particularly noteworthy are the high values for precision, sensitivity, and F1-score, indicating the ability of the proposed feature to correctly classify positive cases and minimize false positives. Overall, both tables affirm that the inclusion of the proposed feature, with careful selection and optimization, significantly improves the predictive accuracy of CVD across different datasets.

## 5 Conclusion and future work

The conclusion of the paper underscores the significant advancements made in the prediction of CVD through the development and application of a *Hybrid Model that integrates LSTM and QNN*. This model, optimized by a novel algorithm, demonstrates exceptional efficacy in handling complex healthcare data, as evidenced by its superior performance metrics over existing models. Notably, the model achieves a remarkable *14.05% improvement in accuracy on Dataset-1 and a 20.7% enhancement on Dataset-2*, with sensitivity metrics that outperform a broad spectrum of current models including SVM, DBN, RNN, DCNN, BiGRU, SMO, SSA, DHOA, and AO. These results not only validate the model’s capability in accurately predicting CVD but also highlight its potential to significantly impact future healthcare practices by providing more precise and reliable diagnoses. Looking forward, the research identifies several areas for potential improvement and expansion, such as refining the optimization algorithm, further tuning the hybrid model, broader evaluation across diverse datasets, exploration of real-time implementation possibilities, and incorporation of additional data sources. These directions aim to further enhance the model’s accuracy and applicability, contributing to the ongoing evolution of predictive healthcare models and ultimately, to the advancement of patient care in cardiovascular diseases.

## Data availability statement

Publicly available datasets were analyzed in this study. This data can be found at: https://archive.ics.uci.edu/dataset/45/heart+disease.

## Author contributions

AD: Conceptualization, Investigation, Methodology, Writing – review & editing. RC: Methodology, Supervision, Writing – review & editing. MA: Formal analysis, Project administration, Writing – review & editing. SD: Conceptualization, Investigation, Writing – original draft. KA: Funding acquisition, Resources, Supervision, Writing – review & editing. UL: Methodology, Project administration, Validation, Writing – original draft.
